# A SYBR Green 1-based *in vitro* test of susceptibility of Ghanaian *Plasmodium falciparum* clinical isolates to a panel of anti-malarial drugs

**DOI:** 10.1186/1475-2875-12-450

**Published:** 2013-12-17

**Authors:** Neils B Quashie, Nancy O Duah, Benjamin Abuaku, Lydia Quaye, Ruth Ayanful-Torgby, George A Akwoviah, Margaret Kweku, Jacob D Johnson, Naomi W Lucchi, Venkatachalam Udhayakumar, Christopher Duplessis, Karl C Kronmann, Kwadwo A Koram

**Affiliations:** 1 Centre for Tropical Clinical Pharmacology and Therapeutics, University of Ghana Medical School, Accra, Ghana; 2 Epidemiology Department, Noguchi Memorial Institute for Medical Research, University of Ghana, Accra, Ghana; 3 US Naval Medical Research Unit No. 3, Cairo, Egypt; 4 Hohoe Health Research Centre, Hohoe, Ghana; 5 Department of Emerging Infectious Diseases Program, US Army Medical Research Unit-Kenya, Kenya Medical Research Institute-Walter Reed Project, Kisumu, Kenya; 6 Division of Parasitic Diseases and Malaria, Center for Global Health, Centers for Disease Control and Prevention, Atlanta, Georgia, USA

**Keywords:** Isolates, *in vitro*, Susceptibility, Inhibition, *Plasmodium falciparum*

## Abstract

**Background:**

Based on report of declining efficacy of chloroquine, Ghana shifted to the use of artemisinin-based combination therapy (ACT) in 2005 as the first-line anti-malarial drug. Since then, there has not been any major evaluation of the efficacy of anti-malarial drugs in Ghana *in vitro*. The sensitivity of Ghanaian *Plasmodium falciparum* isolates to anti-malarial drugs was, therefore, assessed and the data compared with that obtained prior to the change in the malaria treatment policy.

**Methods:**

A SYBR Green 1 fluorescent-based *in vitro* drug sensitivity assay was used to assess the susceptibility of clinical isolates of *P. falciparum* to a panel of 12 anti-malarial drugs in three distinct eco-epidemiological zones in Ghana. The isolates were obtained from children visiting health facilities in sentinel sites located in Hohoe, Navrongo and Cape Coast municipalities. The concentration of anti-malarial drug inhibiting parasite growth by 50% (IC_50_) for each drug was estimated using the online program, ICEstimator.

**Results:**

Pooled results from all the sentinel sites indicated geometric mean IC_50_ values of 1.60, 3.80, 4.00, 4.56, 5.20, 6.11, 10.12, 28.32, 31.56, 93.60, 107.20, and 8952.50 nM for atovaquone, artesunate, dihydroartemisin, artemether, lumefantrine, amodiaquine, mefloquine, piperaquine, chloroquine, tafenoquine, quinine, and doxycycline, respectively. With reference to the literature threshold value indicative of resistance, the parasites showed resistance to all the test drugs except the artemisinin derivatives, atovaquone and to a lesser extent, lumefantrine. There was nearly a two-fold decrease in the IC_50_ value determined for chloroquine in this study compared to that determined in 2004 (57.56 nM). This observation is important, since it suggests a significant improvement in the efficacy of chloroquine, probably as a direct consequence of reduced drug pressure after cessation of its use. Compared to that measured prior to the change in treatment policy, significant elevation of artesunate IC_50_ value was observed. The results also suggest the existence of possible cross-resistance among some of the test drugs.

**Conclusion:**

Ghanaian *P. falciparum* isolates, to some extent, have become susceptible to chloroquine *in vitro,* however the increasing trend in artesunate IC_50_ value observed should be of concern. Continuous monitoring of ACT in Ghana is recommended.

## Background

Malaria, caused by an infection with *Plasmodium falciparum,* is complex and affects a significant number of people living in disease-endemic areas of the world, especially sub-Saharan Africa. According to the World Health Organization (WHO) World Malaria Report, there were about 219 million cases of malaria in 2010 and an estimated 660,000 deaths [1]. Most of these cases occur among children within whom the disease can sometimes present in a severe form, often with devastating consequences. Countries in sub-Saharan Africa, comprising some of the poorly developed nations in the world, bear a major part of the disease burden with at least 90% of the reported deaths [1,2].

In Ghana, malaria is hyper-endemic and remains the most widely diagnosed infectious disease in the country. It is the single most important cause of mortality and morbidity especially among children under five years and pregnant women [3]. The disease is responsible for up to 40% of daily outpatient consultations at hospitals and clinics across the country, accounting for over 23% of deaths among children below the age of five years [4-6]. Early presumptive treatment of febrile illness with chloroquine was the mainstay of malaria control in Ghana until 2005 when there was strong indication of *P. falciparum* resistance to this drug. Reports from drug efficacy study conducted in the country provided strong evidence of the existence of *P. falciparum* isolates that were resistant to chloroquine [7]. Based on this evidence and upon the recommendation of the WHO among others, in 2005 Ghana officially changed from the use of chloroquine to artemisinin-based combination therapy (ACT) as the first choice of anti-malarial drugs for the treatment of uncomplicated malaria. At the moment, ACT recommended by the national malaria control programme (NMCP) of Ghana is artesunate–amodiaquine (AA), with artemether-lumefantrine (AL) and dihydoartemisinin-piperaquine (DHAP) as alternatives. It must be emphasized that in the absence of either an effective vaccine or good alternative anti-malarial drugs to ACT, the emergence and spread of artemisinin-resistant parasites would be devastating. Although no resistance to combination therapy has yet been reported in Ghana, it is important that these drugs are closely monitored for early detection of reduced parasite susceptibility, especially as reports have appeared of *P. falciparum* isolates with decreased response to artemisinin in other parts of the world [8].

*In vitro* test of *P. falciparum* susceptibility to anti-malarial drugs is one of the important tools that can be used to monitor the efficacy of anti-malarial drugs, as results of parasite responses to drugs may show early trends in changes to susceptibility to the tested drugs and may serve as an early warning system of resistance development in the parasite population [9]. Although *in vivo* drug efficacy studies remain the ‘gold standard’ for assessment of anti-malarial drug resistance, its use is limited because it is prohibitively expensive [10]. Molecular marker determination can also be used to identify the single-nucleotide polymorphisms commonly associated with drug resistance in malaria parasites; however, the methods require specialized equipment, which are costly and the assay is difficult to conduct in the field in real time [11]. Additionally, these markers are not well described for the artemisinins. With the low cost involved in carrying out the assay and the rapidity with which it could be conducted, the *in vitro* drug sensitivity test has become a strong choice for assessing anti-malarial drug efficacy in disease-endemic areas. The test is not affected by host-confounding factors such as immunity, compliance, concomitant infections, re-infection/recrudescence, poor drug absorption, etc. [12,13]. The recently described SYBR Green 1 *in vitro* assay for assessment makes performing the assay easier and precise [14].

Since Ghana officially changed its malaria treatment policy in 2005, there has been no major nationwide *in vitro* assessment of parasites responses to anti-malarial drugs. In order to determine if the change in policy has significantly affected the susceptibility of the parasites to anti-malarial drugs, this study was carried out to measure the responses of clinical isolates of *P. falciparum* to anti-malarial drugs and compare the outcome with baseline data generated from a similar survey conducted in 2004 [15]. The *in vitro* susceptibility of *P. falciparum* isolates to a panel of anti-malarial drugs was assessed using the newly developed SYBR Green 1-fluorescent-based method. The panel of 12 anti-malarials includes the nationally recommended anti-malarial drugs for treatment of uncomplicated malaria in Ghana, drugs used for malaria prevention in travellers, and the previous first-line drug in the country, chloroquine. Cross-resistance between drugs from the same chemical class or between drugs with similar modes of action, and correlations between susceptibility to different drug classes were also determined and discussed.

## Methods

### Study sites

**Figure 1:**
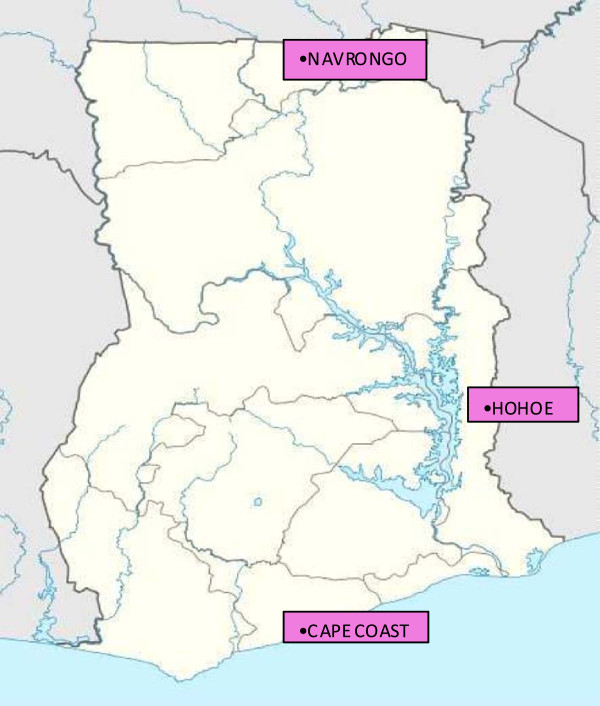
**Map of Ghana.** A map of Ghana showing the sentinel sites used for the study. The sites are located in three unique ecological zones in Ghana namely Coastal savannah (Cape Coast), Forest (Hohoe) and Guinea savannah (Navrongo). All sites are high transmission areas, but differ from each other in the seasonal variation in malaria rates.

Three sentinel sites, Cape Coast, Hohoe and Navrongo, representing three distinct eco-epidemiological zones in Ghana were selected for the study (Figure 1). Cape Coast (5°.07’N, 1°.11’W) is the capital of the Central region of Ghana. It has a coastline of about 13 km and is about 150 km west of Accra (the capital city of Ghana). Vegetation in this area is mainly coastal savannah. Generally, there are two rainy seasons in this region with the peak of the major season occurring in June. Numerous rivers and streams in this area end up in a lagoon, creating a vast wetland. The presence of these water bodies allows vector breeding throughout the year, hence malaria transmission in this area is perennial. Hohoe (7º99N, 0°289E) is the capital of Hohoe municipality and lies in the middle belt of the country, about 220 km north east of Accra. This area with a vegetation of semi-deciduous forest has two rainy seasons a year, the major one occurring in May-June. Malaria transmission in this site is perennial with a moderate seasonal peak occurring after the major rains in June. Navrongo (10°549N, 1°69W), is the capital of Kassena-Nankana district. It is about 865 km north of Accra and lies in the Guinea savannah area in the Upper East region of Ghana. It receives all of its annual rainfall between May and October. The presence of a large reservoir in the district, created to irrigate the land for farming, allows mosquitoes to breed throughout the year. As a result, malaria is perennial but with marked seasonal peak transmission occurring between June and November each year.

### Study population and recruitment

During the main malaria seasons in 2012, children aged six months to nine years presenting to clinics at the study sites with fever or history of fever within the previous 24 hours were screened for inclusion in the investigation. To be included in the study the child had to have documented *P. falciparum* mono-infection at a count ranging between 1,000 and 250,000 per μl. Detailed information on the study was made available to the parents or guardians of potential participants and they were encouraged to ask questions about any aspect of the study that was unclear to them. A child was only enrolled if the parents or guardians gave their informed consent. Sixty-three (63) patients were recruited from each of the three sites to participate in the study. Permission to carry out this work and ethical clearance were obtained from the Institutional Review Board (IRB) of the Noguchi Memorial Institute for Medical Research (NMIMR), Ghana. This study also received ethical approval from the US Naval Medical Research Unit No. 3 (NAMRU-3) IRB, Cairo, Egypt.

### Sample collection

Prior to treatment, 2 ml of blood were aseptically collected from each participant into a tube containing citrate phosphate dextrose-adenine (CPD-Adenine) and transported to the laboratory for the *in vitro* drug test within 24 hours. The blood was diluted 20× with complete RPMI 1640 (Gibco, UK) and used for the assay.

### *In vitro* test of susceptibility of *Plasmodium falciparum* to anti-malarial drugs

#### ***Preparation of media, drugs dilutions and test plates***

Incomplete RPMI 1640 culture media supplemented with hypoxanthine and glucose were prepared as previously described [14]. Complete RPMI 1640 contains NaHCO_3_ and Albumax (Invitrogen). All drugs used in this study were supplied by the World Wide Antimalarial Resistance Network (WWARN), Centers for Disease Control and Prevention (CDC), USA and Walter Reed Army Institute of Research (WRAIR), Kisumu, Kenya. The panel of 12 drugs tested in this study included: amodiaquine, artesunate, artemether, atovaquone, chloroquine, dihydroartemisinin, doxycycline, lumefantrine, mefloquine, piperaquine, quinine, and tafenoquine. Five ml of stock solutions at 1 mg/ml were prepared for each anti-malarial drug. Amodiaquine, quinine, mefloquine, and artemisinin were dissolved in 70% ethanol and lumefantrine and doxycycline in 100% dimethyl sulphoxide (DMSO). Chloroquine was first dissolved in 1.5 ml deionized water after which the solution was made up to 5 ml with absolute ethanol. The drug solutions prepared were used immediately or stored at -80°C for not longer than one month before use. Stock solutions were further diluted in complete RPMI 1640 to the desired starting concentrations after which two-fold serial dilution was performed in 96-well tissue culture plate to generate ten concentrations for the *in vitro* drug test. The concentration range for the drugs (ng/ml) and molecular weights (g/mol), which was later used to convert to nM of the test drug concentration were, respectively: amodiaquine (0.78-200 ng/ml, 464.51), artesunate (0.78-200 ng/ml, 384.4), artemether (0.78-200 ng/ml, 298.37), atovaquone (0.195-50 ng/ml, 366.84), chloroquine (7.8-2,000 ng/ml, 515.86), dihydroartemisinin (0.78-200 ng/ml, 284.35), doxycycline (390.6-100,000 ng/ml, 512.94), lumefantrine (0.78-200 ng/ml, 528.94), mefloquine (1.9-500 ng/ml, 414.77), piperaquine (15.6-4,000 ng/ml, 999.55), quinine (15.6-4,000 ng/ml, 321.41) and tafenoquine (19.5-5,000 ng/ml, 463.49). Once pre-dosed with the anti-malarial drugs, the plates were kept at 4°C until use. Test plates were used within three days after preparation.

### Drug sensitivity testing

Two ml of blood collected from the patients was diluted 20× with complete RPMI 1640 and 100 μl was added to each well of the pre-dosed test plate, starting with the lowest concentration of drug and then progressively to higher ones. Wells containing no drug but the diluted patient’s blood was included on each plate. The plate was placed in a modular incubator chamber and gassed (gas contains 92.5% N_2_, 5.5% CO_2_, 2% O_2_). The chamber was placed in an incubator set at 37ºC for 72 hours. Laboratory reference clones, 3D7, regarded as chloroquine sensitive and DD2 classified as chloroquine resistant, were assayed periodically as internal control.

Assessment of the outcome of the *in vitro* drug test was done using the SYBR Green1 method previously described by Johnson and colleagues [14]. In brief, after 72 hours of incubation, the test plate was removed and 100 μl Malaria SYBR Green 1 fluorescent (MSF) lysis buffer containing SYBR Green was added to each well and mixed thoroughly by gently tapping on the plate. The plate was covered with aluminium foil and incubated at room temperature in the dark for at least two hours. Fluorescence was then read on the prototype micro titer plate reader (MTPR) (QIAGEN).

### Data analysis

The concentration of anti-malarial drug inhibiting parasite growth by 50% (IC_50_) for each drug was estimated from a dose response curve by non-linear regression analysis using an online program [16] previously described by the groups of Le Nagard and Kaddouri [17,18]. The program generated IC_50_ estimates with associated 95% confidence intervals (CI). Estimated values with insufficient precision based on the CI were discarded. Geometric mean (GM) IC_50_ was calculated for each drug per sentinel site and a pooled national GM IC_50_ valued was also determined. The use of GM was to minimize the effects of outlier values. In order to check for evidence of cross resistance, a Spearman’s Rank Order correlation was run to determine the relationship between drugs with similar modes of action or for those belonging to the same chemical class. A p-value of 0.05 was considered indicative of a statistically significant relation. Scatter graph and bar charts were used to present some of the results.

## Results

Majority of the children clinically diagnosed with malaria and confirmed by microscopy to have an infection with *P. falciparum* qualified to participate in the study. Sixty three clinical isolates were collected within one month per site. Over 85% of the 189 *P. falciparum* clinical isolates collected from the three selected sentinel sites were successfully cultured and their susceptibilities to the test anti-malarial drugs determined.

The outcome of the test of susceptibilities of clinical isolates of *P. falciparum* collected from three sentinel sites in Ghana is shown in Additional file 1: Table S1. When the values for all the study sites were pooled, the GM IC_50_ values determined for the country were 1.60, 3.80, 4.00, 4.56, 5.20, 6.11, 10.12, 28.32, 31.56, 93.60, 107.20, and 8952.50 nM for atovaquone, artesunate, dihydroartemisin, artemether, lumefantrine, amodiaquine, mefloquine, piperaquine, chloroquine, tafenoquine, quinine, and doxycycline, respectively. Extremely high IC_50_ values were observed for some of the anti-malarial drugs; for example, values of 1441.8 nM, 109.4 μM, 125.9 nM and 6381.9 nM which are far above the threshold IC_50_ values discriminative for resistance were measured for chloroquine, doxycycline, mefloquine, and quinine, respectively. Generally, the isolates from Cape Coast appeared to exhibit higher IC_50_ values to most of the drugs compared to those from the other sites.

**Figures 2:**
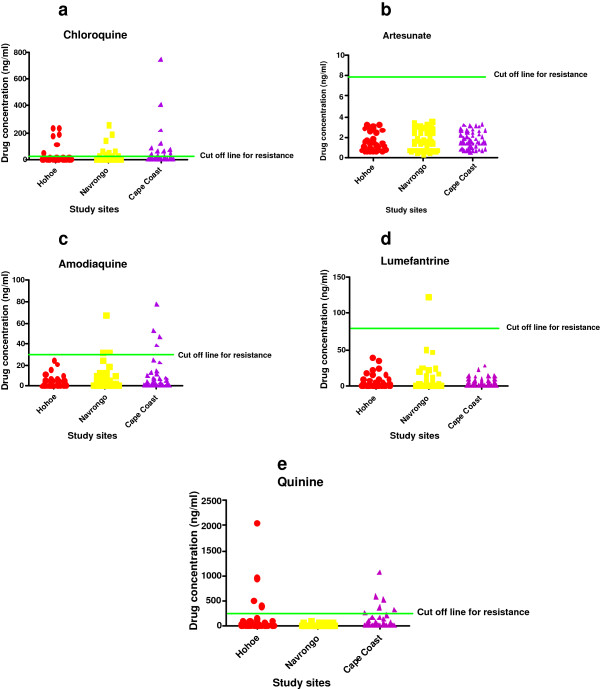
**Scatter plots of GMIC50 values determined for test antimalarial drugs. a**-**e** are Plots of IC50 values determined from test of susceptibility of P. falciparum clinical isolates to some popular anti-malarial drugs used in Ghana. The isolates were collected from three sentinel sites in the country shown as red for Hohoe, yellow for Navrongo and purple for Cape Coast. The olive green lines on each graph indicate the IC50 threshold points discriminative for resistance to the drug.

A snapshot of a scatter plot of IC_50_ values for six of the popular anti-malarial drugs used in Ghana is shown in Figure 2 (a-e). The percentage of the isolates that were resistant for each of the anti-malarial drugs tested per site based on published threshold IC_50_ values discriminative for resistance is also shown in Additional file 1: Table S1. The literature IC_50_ cut-off value indicative of resistance used in this study were chloroquine, 100 nM [19-21]; mefloquine, 30 nM [19,21,22]; amodiaquine, 80 nM [20-22]; lumefantrine, 150 nM [21,23]; doxycycline, 35 μM [21]; artesunate, 20 nM [21]; quinine, 800 nM [20,22]; dihydroartemisinin, 12 nM [21] and artemether, 30 nM [21,24]. Cut-off resistant values for piperaquine and tafenoquine were not available in the literature. It is worth noting that prior to the emergence of atovaquone resistance, Gay and colleagues published a cut-off value of 5–7 nM for resistance [25]. However, upon the emergence of *P. falciparum* resistance to atovaquone, the group of Musset revised the cut-off to 1,900 nM after investigations using resistant phenotype [26]. For the drugs with known literature threshold IC_50_ values indicative of resistance, the determined levels of resistance recorded in this study were 13.5, 16.6, 3.7, 0.7, 23.7, 0, 7.1, 0, 0, and 0% for chloroquine, mefloquine, amodiaquine, lumefantrine, doxycycline, artesunate, quinine, dihydroartemisinin, artemether, and atovaquone, respectively.

Although the radio-isotopic method was used in determining the cut-off values indicative of resistance, it must be emphasised that the IC_50_ values generated with the Sybr Green 1fluorescence method is reported to be comparable. Smilkstein and co-workers reported that the IC_50_ of standard anti-malarial drugs determined with both radio-isotopic and Sybr Green methods were similar or identical [27]. Although the group of Johnson also reported a similar observation, however the group admitted that a statistically significant difference exist between IC_50_ values generated between the two assays [13]. The group however found the sensitivity index to be the same for the two methods, suggesting that although statistically significant differences do exist between the two assays, they are likely not biologically significant[13].

**Figure 3:**
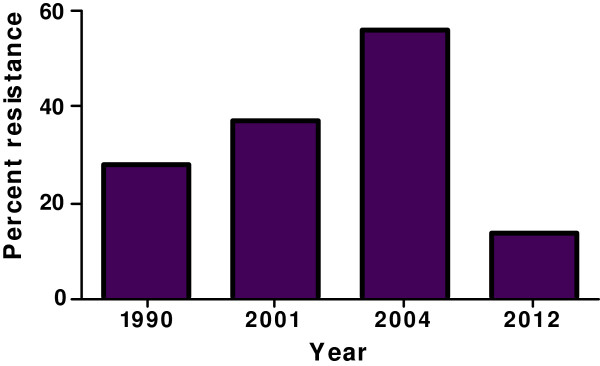
**Trends in chloroquine resistance *in vitro *in Ghana.** Trends in resistance of Ghanaian *P. falciparum* isolates to chloroquine *in vitro* from 1990 through 2012 [15,28,29]. The number of isolates assessed was 195, 64, 57, and 141 for the year 1990, 2001, 2004 and 2012 respectively. NB: the current report is shown in the chart as 2012.

**Figures 4:**
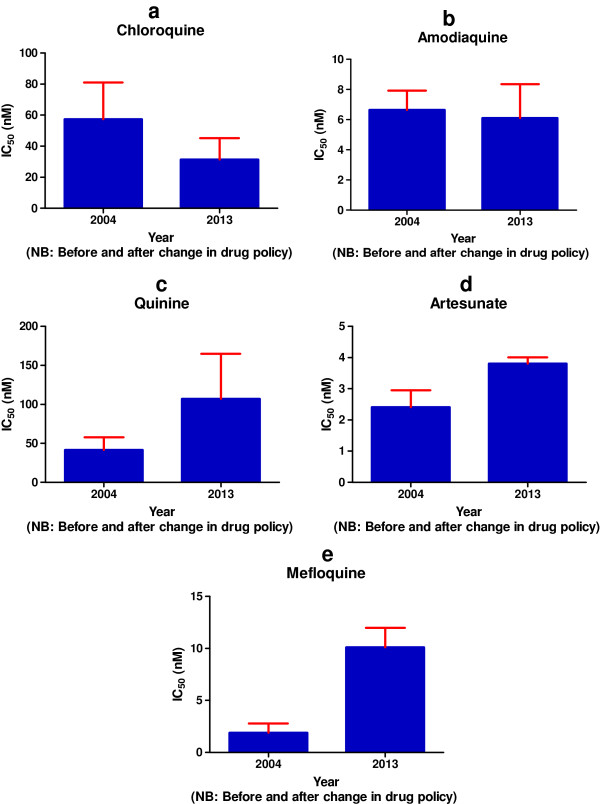
**Comparison between GMIC50 values of antimalarial drugs.** The GM IC50 values of some selected anti-malarial before (2004) and eight years after (2012) the change in malaria treatment policy in Ghana were compared. The comparison is shown in **a**-**e** of the figure for chloroquine, amodiaquine, quinine, artesunate and mefloquine respectively. The error bars are the standard error of the mean.

Figure 3 shows the trend in *in vitro* responses of Ghanaian *P. falciparum* isolates to chloroquine between 1990 and 2012. Resistance to chloroquine *in vitro* increased from 1990 to an all-time high in 2004 and decreased significantly in 2012. Figure 4 (a-e) shows the comparison of IC_50_ value of some of the popularly used anti-malarial drugs in Ghana before the change in treatment policy (2004) and the current report (2012). There was a drastic reduction in IC_50_ values for chloroquine determined in 2012 compared with that of 2004: more than 50% decrease in the pooled national GM IC_50_ values between the two dates. Compared to the data from the 2004 survey, the current results showed a moderate increase in GM IC_50_ value for artesunate and a high increase for quinine and mefloquine.

The level of correlation between the IC_50_s of some of the anti-malarial drugs studied per sentinel site is shown in Additional file 2: Table S2. A p-value of <0.05 was considered as the threshold indicative of a statistically significant correlation. Significant correlation was found among the following pairs of drugs: amodiaquine *versus* quinine (at Cape Coast); artemether *versus* dihydroartemisinin (at Cape Coast and Hohoe); chloroquine *versus* quinine (at Hohoe); amodiaquine *versus* mefloquine (at Hohoe); mefloquine *versus* quinine (at Navrongo).

To ensure that the reagents or drugs used in this study maintained their quality throughout the study period, 3D7 and DD2 clone of *P. falciparum* was tested fortnightly against known drugs and the IC_50_ values obtained compared with universally acceptable values for the drugs.

## Discussion

*In vitro* assessment of the susceptibility of malaria parasites to drugs remains an important component of anti-malarial drug efficacy surveillance. Since this method is largely independent of clinical factors, it provides information that complements clinical assessment of drug efficacy. The SYBR Green1 method of assessing the outcome of the *in vitro* drug test was revalidated and used to assess the responses of *P. falciparum* clinical isolates to a panel of 12 anti-malarial drugs in Ghana. To the best of knowledge, this is the first use of the SYBR Green 1 method in Ghana and the reported assertion that it is easy to use, reliable and cheaper could be affirmed. All the components of ACT currently used in Ghana as well as quinine and the previous first-line anti-malarial drug, chloroquine were among the test drugs.

Compared with findings from a similar survey conducted in 2004 [15], the overall resistance to chloroquine determined in this study dropped drastically from 56 to 13.5%. A pooled national GM IC_50_ of chloroquine was also observed to have decreased by more than 50% compared to the 2004 value. These observations are consistent with reports from East African countries, Malawi and Kenya, indicating the return of chloroquine-sensitive isolates following a similar official withdrawal of the drug [30-32]. It also confirms an observation made in a study conducted in France using isolates collected from returning visitors from Senegal, Mali, Ivory Coast, and Cameroon [33]. The large improvement in the efficacy of chloroquine observed in the present study is important as it seems to reflect the real situation on the ground. Indeed, this gives credence to recent finding in Ghana indicating a significant decline in the prevalence of *P. falciparum* chloroquine-resistant transporter gene (*pfcrt) codon*76 mutant allele (T76) and *P. falciparum* multidrug-resistant gene (*pfmdr1) codon*86 mutant allele (Y86) in the country [34]. Prevalence of *pfcrt* T76 mutation has been associated with clinical chloroquine resistance and represents a good indicator of the parasite’s intrinsic resistance to the drug [35,36]. Additionally, single nucleotide polymorphisms (SNPs) in the *pfmdr*1 on chromosome 5 which encodes a P-glycoprotein homologue-1 multi-drug resistant transporter is associated with enhanced efflux of the drug from resistant parasites [37]. Association of chloroquine resistance with *pfmdr*1 Y86 has been reported in many genetic studies including one carried out in Ghana by the group of Koram [38,39]. Eight years have elapsed since chloroquine was replaced with ACT as the first-choice anti-malarial drug in Ghana. It is, therefore, likely that the withdrawal of chloroquine from use over these years might have caused a decrease in drug pressure with a consequent decline of chloroquine-resistant strains.

Currently, AA is one of the officially recommended ACT selected for treatment of uncomplicated malaria in Ghana. The combination is also used for the treatment of uncomplicated malaria in the second and third trimester of pregnancy and is recommended for the assisted home management of malaria in Ghana [40]. Although all the isolates tested in this study appear to be sensitive to artesunate, of grave concern is the increased pooled national GM IC_50_ value measured in this study compared with that of 2004. This observation suggests an emerging population of malaria parasites with tolerance for higher concentrations of artesunate. One explanation could be selective drug pressure since ACT is now the first line of therapy for uncomplicated malaria. However, another possible explanation may be that artesunate is being used inappropriately in the country thus facilitating the development of ‘low level resistance’ by malaria parasites. Published data by Kwansa-Bentum and colleagues confirms the indiscriminate use of artesunate monotherapy for the treatment of malaria in Ghana [41]. The consequences of continuation of this practice are obvious. There is the need to adhere strictly to the current national treatment guidelines which are in conformity with the WHO guidelines as endorsed by the World Health Assembly [42-44]. Recently, a new method for the assessment of the response of *P.falciparumin* to the artemisinins *in vitro* was developed. This is in response to reports suggesting that artemisinin resistant parasites tolerate high levels of the drug by exiting dormancy and resuming growth at a greater rate than susceptible parental strains [45]. This situation makes it difficult to evaluate the *in vitro* activity of the artemisinin derivatives by standard tests. In the light of this, a new method called ‘the Ring-stage Survival Assay (RSA)’ which is supposed to adequately measure *P. falciparum* resistance to the aremisinins was developed and published by Witkowski and co-workers [46]. With regard to amodiaquine, there was no significant change in the GM IC_50_ value determined in this study compared to the 2004 value. However, a few of the *P. falciparum* isolates were observed to be resistant to the drug *in vitro*. Amodiaquine is chemically related to chloroquine, and it is not extensively used in Ghana for monotherapy. The high susceptibility of the parasite to amodiaquine observed in the present study might be explained both by the decline in chloroquine-resistant isolates discussed earlier and/or the switch from amodiaquine monotherapy to AA combination therapy: the combination might have offered protection to amodiaquine and precipitated the improvement or stability of amodiaquine and chloroquine susceptibility. The stability in potency justifies the continuous use of amodiaquine as a component of the official ACT.

Artemether-lumefantrine combination was recommended as an alternative for the treatment of uncomplicated malaria in Ghana following reports of adverse reaction to AA [47]. All the *P. falciparum* isolates tested in this study were susceptible to artemether with an overall national GM IC_50_ value of 4.5 nM. The isolates also responded to lumefantrine with a pooled national GM IC_50_ value of 5.2 nM. Based on the literature cut-off for resistance, only one isolate showed resistance to lumefantrine. There is no baseline *in vitro* data on these two drugs in Ghana hence the changes that might have occurred in their efficacy since the change in the treatment policy could not be discussed. However, compared with published data from studies conducted in other countries, the IC_50_ values of the drugs measured in the present study are much lower.

Combination of dihydroartemisinin and piperaquine is another form of ACT recommended for the treatment of uncomplicated malaria in Ghana. All the isolates assessed in this study were sensitive to dihydroartemisinin. Since the former is the active metabolite of artesunate, the result was not surprising. No correlation indicative of cross-resistance was found between artesunate and dihydroartemisinin. Resistance level of piperaquine could not be ascertained in this study due to the unavailability of literature cut-off IC_50_ value indicative of resistance to the drug.

A geometric mean IC_50_ value of 107.2nM determined for quinine is more than double that reported in 2004. Furthermore, unlike in 2004, some of the isolates tested in this study were resistant to the drug. Quinine is an important anti-malarial drug in Ghana as it remains the drug of choice for the management of complicated malaria and in the event of ACT treatment failure. Oral quinine or a combination of oral quinine and clindamycin is also the recommended drug for the management of uncomplicated malaria during the first trimester of pregnancy in Ghana [47]. Since this drug is not used on a regular basis in Ghana, a clear explanation for the decrease in parasite susceptibility to the drug observed *in vitro* is not easy to come by. However, it is noteworthy that in surveys of drug quality in sub-Saharan Africa, quinine has often been found to be sub-standard, including samples with low concentrations of active ingredient [48]. The use of sub-standard drug is likely to jeopardize the efficacy of the anti-malarial drug.

Mefloquine and atovaquone are recommended drugs for non-immune travellers for chemoprophylaxis. Even though the pooled GM IC_50_ determined in this study for mefloquine was far below the cut-off value discriminative for resistance, about 16.6% of the *P. falciparum* isolates assessed were resistant to the drug. Compared with the level observed in the 2004 study, the IC_50_ measured here is over five-fold increase. Mefloquine, which belongs to the amino-alcohol class, is not used much in Ghana, hence drug pressure could not explain the increase in resistance. However, since some *in vitro* studies have demonstrated an inverse relationship between the responses of chloroquine and the amino-alcohols [49,50] this phenomenon could best explain the observation. All the *P. falciparum* isolates tested in this study were sensitive to atovaquone.

Tafenoquine, an 8-aminoquinoline anti-malarial drug, which was recently introduced to the world market, represents a potential alternative to standard agents for the prevention and radical cure of malaria [51]. In the absence of an available literature cut-off value indicative of resistance, the GM IC_50_ value of 93.6 nM obtained in this study was compared with those determined in other countries. The IC_50_ values reported from other countries range from 0.9 to 9.7 μM in Djibouti, 0.6 to 33.1 μM in Gabon, and 0.5 to 20.7 μM in Senegal [52]. Compared with these values, the pooled national GM IC_50_ value of 93.6 nM determined in this study is relatively low and implies that Ghanaian *P. falciparum* isolates are highly sensitive to the drug.

The anti-malarial activity of tetracycline and its derivatives has been demonstrated *in vitro* and *in vivo*[53-55]. Currently doxycycline is recommended as chemoprophylaxis to non-immune travellers to some disease-endemic countries of the world. Doxycycline is also used generally as an antibiotic for the management of a variety of infection such as Lyme disease, acne, urinary tract infections, and pneumonia, among others. However, information on its use in Ghana both as an anti-malarial drug or antibiotic is scanty. In the present study, doxycycline had the least effect on the clinical isolates tested, showing a resistance level of above 20%. The observed high resistance to the drug could be explained in terms of its mechanism of action. A study by Dahl and Rosenthal showed that the pharmacological concentration of the drug was relatively inactive against the parasites initially but exerted a delayed death effect, in which the progeny of treated parasites failed to complete erythrocytic development [56]. The report further indicated that the drug does not alter the distribution of apicoplasts in developing parasites but affects their progeny. This implies that a longer period of exposure is required to achieve maximum effect of doxycycline. Since in the present study the parasites were cultured for only 72 hours, the duration of the culture may be insufficient and could be the reason for the slow anti-malarial action of doxycycline observed in this study. An extended time of incubation to 96 hours would have ensured adequate effect of the drug on the parasite and would have presented a better picture of the Ghanaian *P. falciparum* isolate’s susceptibility to doxycycline.

An important observation made in this study was that the *P. falciparum* isolates from Cape Coast exhibited higher IC_50_ values compared to those from the other sites. Environmental and socio-economic factors could be possible reasons for this observation. The site in Cape Coast receives most of its clients from communities with poor infrastructural development. The presence of stagnant water in these communities might contribute greatly to mosquito breeding and an increase in malaria transmission. This situation is likely to lead to increased anti-malarial use in this area. Indeed, an unpublished investigation by the group of Johnson Boampong (University of Cape-Coast, Ghana) confirmed the indiscriminate use of anti-malarial drugs in the study area (Kwame Asare Kumi, pers comm). This practice is likely to lead to increased drug pressure with a consequent selection and nurturing of resistant parasites.

Cross-resistance could be said to occur when a drug confers resistance to other drugs that have similar mode of action or belong to the same chemical group. Cross-resistance may complicate anti-malarial drug resistance, and its existence is worth investigating. A positive correlation between the responses to two anti-malarial drugs suggests an *in vitro* cross-resistance but not necessarily confer cross-resistance *in vivo.* In the present study, the existence of cross-resistance among some of the test drugs was ascertained. A positive correlation was found between the IC_50_ values for: amodiaquine and quinine, artemether and dihydroartemisinin, chloroquine and quinine, amodiaquine and mefloquine, and mefloquine and quinine. All the observed significant correlations were limited to one site except that between artemether and dihydroartemisinin where it was observed in two of the three sites surveyed. It is worth noting that a significant correlation between two drugs tested *in vitro* doesn’t necessary mean a cross resistance exist among them. For a correlation to imply that two compounds share common mechanisms of action or resistance, which could induce cross-resistance, the coefficient of determination (r^2^) must be high. In this study, the r^2^ values of the drugs showing significant correlation were too low to suggest a strong cross-resistance between them. Contrary to expectation, no positive correlation was observed between artesunate and artemether or dihydroartemisin. A possible explanation for this observation could be the usage of a single ACT in the study areas: artesunate amodiaquine combination is the most widely used ACT in these communities. Significant correlation between some of these drugs may be explained in part by close resemblance in chemical structures. It must be emphasized that clinical and epidemiological significance or implications of the correlation between some of the anti-malarial drugs observed in this study will be difficult to decipher.

Delayed clearance of *P. falciparum* isolates with ACT has recently been reported [8]. The emergence of *P. falciparum* resistance to artemisinin derivatives is of urgent public health concern, which could considerably slow down the global effort to reduce the malaria burden. In the absence of an effective malaria vaccine, steps must be taken to protect the artemisinin derivatives. In the light of this, the WHO has launched the Global Plan for Artemisinin Resistance Containment (GPARC) aimed at avoiding the spread of resistance from the area of first report of resistance to other disease-endemic zones [57]. In Ghana, various measures have been taken by the NMCP to avoid the emergence of drug resistance to ACT; sentinel sites have been set up across the country to monitor the efficacy of ACT. However, with the observations made in this study, the need to adopt a more aggressive approach must be considered. The NMCP needs to launch a more vigorous national campaign against improper use of the artemisinin derivatives. Equally important is drug quality: steps must be taken to eliminate counterfeit ACT and reduce sub-standard manufacturing with lower concentration of artemisinin content. The entire pharmaceutical distribution modes and drug supply chains that impact directly on drug use must be purged to ensure the supply of good quality drugs and the total enforcement of the ban on certain anti-malarial drugs such as chloroquine, or cessation of practices such as the use of the artemisinin derivatives as monotherapy. With the validation and subsequent use of the SYBR Green method in Ghana, continuous assessment of the susceptibility of *P. falciparum* to anti-malarial drugs in the country must be encouraged in order to make available to the NMCP supportive data that will allow prediction of emerging resistant strains of parasites in the country.

## Conclusion

Given the lack of robust molecular markers predictive of anti-malarial resistance for the artemisinins and the huge cost in conducting *in vivo* efficacy study, *the in vitro method* of assessment of the artemisinins and other anti-malarial drugs is warranted. The *in vitro* method was successfully used to assess the sensitivity of Ghanaian *P. falciparum* isolates to 12 anti-malarial drugs. Although frank resistance to artesunate was not observed, a concerning trend of increasing GMIC_50_ since the introduction of ACT was noticed. This situation warrants continuous monitoring of ACT. On the other hand, chloroquine appears to have regained a greater proportion of its efficacy after being out of use as first-line drug for eight years.

## Competing interests

The authors have no competing interest to declare. None of the authors received any remuneration for this work.

## Authors’ contributions

KCK, NBQ, NWL, VU and KAK conceived the idea and worked with BA, NOD, JDJ, CD, and MK on the design and data acquisition. NBQ, GAA, RA, MK, NOD, BA and LQ coordinated the field or laboratory work. NBQ drafted the manuscript. All authors participated in the revisions of the manuscript and gave approval for the final version for publication.

## Supplementary Material

Additional file 1: Table S1 *In vitro* drug susceptibility of *Plasmodium falciparum* isolates to 12 anti-malarial drugs. The drug sensitivities of the isolates collected from clinics in three sentinel sites in Ghana were assessed using the SYBR Green1 method and the results presented below. Proportion of *P. falciparum* clinical isolates per sentinel site that were resistant to the anti-malarial drugs tested, based on literature cut-off IC_50_ values (last column) is also shown. Click here for file

Additionalo file 2: Table S2 Cross-resistance between test anti-malarial drugs. Degree of correlation (r) between the IC_50_s of some of the test anti-malarial drugs per sentinel site using Spearman’s rank order correlation. The statistical significance of the correlation is also indicated. A p-value of <0.05 was considered indicative of statistically significant correlation. Click here for file
