# Supplementary material for: A SYBR Green 1-based in vitro test of susceptibility of Ghanaian Plasmodium falciparum clinical isolates to a panel of anti-malarial drugs
Source: Malar J. 2013 Dec 17;12:450. doi: 10.1186/1475-2875-12-450 (PMC3878558; doi:10.1186/1475-2875-12-450)
Supplement: Additional file 1: Table S1 — In vitro drug susceptibility of Plasmodium falciparum isolates to 12 anti-malarial drugs. The drug sensitivities of the isolates collected from clinics in three sentinel sites in Ghana were assessed using the SYBR Green1 method and the results presented below. Proportion of P. falciparum clinical isolates per sentinel site that were resistant to the anti-malarial drugs tested, based on literature cut-off IC50 values (last column) is also shown. [file 1475-2875-12-450-S1.docx]

**Table 1 *In vitro* drug susceptibility of *Plasmodium falciparum* isolates to 12 anti-malarial drugs**

The drug sensitivities of the isolates collected from clinics in three sentinel sites in Ghana were assessed using the SYBR Green1 method and the results presented below. Proportion of *P. falciparum* clinical isolates per sentinel site that were resistant to the anti-malarial drugs tested, based on literature cut-off IC_50_ values (last column) is also shown

| **Drug** | **Geometric mean IC_50_ values (nM) of anti-malarial drugs per study site (range)** | | | **Percent parasite resistance per study site** | | | **Cut-off for resistance** |
| --- | --- | --- | --- | --- | --- | --- | --- |
|  | **Hohoe** | **Navrongo** | **Cape Coast** | **Hohoe** | **Navrongo** | **Cape Coast** |  |
| **Chloroquine** | 30.91  (9.7-467.18) | 28.98  (12.02-500.38) | 36.85  (11.28-1,441.78) | 10.5% | 11.1% | 19% | >100 nM |
| **Mefloquine** | 12.24  (3.6-102.91) | 8.77  (3.9-125.87) | 9.76  (3.04-48.82) | 23.1% | 12.2% | 14.6% | >30 nM |
| **Amodiaquine** | 5.20  (1.29-52.24) | 6.10  (1.16-144.26) | 7.7  (1.03-167.63) | 0% | 2.3% | 9.3% | > 80 nM |
| **Lumefantrine** | 6.48  (1.13-75.22) | 4.80  (1.23-232.66) | 4.71  (1.17-52.73) | 0% | 2.1% | 0% | > 150 nM |
| **Doxycycline** | 8349.9  (628.30-109,365.75) | 7,835  (505.67-105,202) | 10,672.5  (650.52-10,6564) | 17.5% | 23.6% | 30% | > 35 μM |
| **Piperaquine** | 29.69  (12.4-230.1) | 23.91  (11.66-82.9) | 33.22  (10.5-633.4) | - | - | - | NA |
| **Artesunate** | 3.59  (1.79-8.50) | 3.67  (1.07-9.36) | 4.06  (1.38-8.68) | 0% | 0% | 0% | >20 nM |
| **Qunine** | 126.32  (41.23-6,381.97) | 73.59  (39.80-282.13) | 136.49  (33.82-3,306.77) | 10.2% | 0% | 11.3 % | > 800 nM |
| **Dihydroartemisinin** | 3.58  (2.00-10.74) | 3.82  (2.32-10.6) | 4.49  (1.51-10.21) | 0% | 0% | 0% | >12 nM |
| **Artemether** | 5.12  (2.21-10.41) | 4.25  (1.94-10.32) | 4.25  (1.64-8.88) | 0% | 0% | 0% | >30 nM |
| **Tafenoquine** | 113.99  (28.77-2,719.5) | 47.29  (26.50-140.47) | 160.19  (25.62-4,898.67) | - | - | - | NA |
| **Atovaquone** | 2.72  (0.49-67.77) | 1.16  (0.40-11.66) | 1.67  (0.46-74.60) | 0% | 0% | 0% | >1,900 nM |

NA=Not available in literature
