# Supplementary material for: A SYBR Green 1-based in vitro test of susceptibility of Ghanaian Plasmodium falciparum clinical isolates to a panel of anti-malarial drugs
Source: Malar J. 2013 Dec 17;12:450. doi: 10.1186/1475-2875-12-450 (PMC3878558; doi:10.1186/1475-2875-12-450)
Supplement: Additionalo file 2: Table S2 — Cross-resistance between test anti-malarial drugs. Degree of correlation (r) between the IC50s of some of the test anti-malarial drugs per sentinel site using Spearman’s rank order correlation. The statistical significance of the correlation is also indicated. A p-value of <0.05 was considered indicative of statistically significant correlation. [file 1475-2875-12-450-S2.docx]

**Table 2 Cross-resistance between test anti-malarial drugs**

Degree of correlation (r) between the IC_50_s of some of the test anti-malarial drugs per sentinel site using Spearman’s rank order correlation. The statistical significance of the correlation is also indicated. A p-value of <0.05 was considered indicative of statistically significant correlation

| **Drug pair** | **Cape Coast** | | | **Hohoe** | | | **Navrongo** | | |
| --- | --- | --- | --- | --- | --- | --- | --- | --- | --- |
|  | **r** | **p-value** | **indication** | **r** | **p-value** | **Indication** | **r** | **p-value** | **indication** |
| **Chloroquine *vs* amodiaqune** | 0.2013 | 0.2462 | NS | 0.2349 | 0.1678 | NS | -0.0758 | 0.6418 | NS |
| **Chloroquine *vs* mefloquine** | 0.1621 | 0.3835 | NS | 0.2123 | 0.2688 | NS | 0.2947 | 0.0686 | NS |
| **Chloroquine *vs* quinine** | 0.2859 | 0.0737 | NS | 0.468 | 0.0069 | S | 0.2027 | 0.1766 | NS |
| **Mefloquine *vs* quinine** | -0.06098 | 0.7319 | NS | -0.0491 | 0.7964 | NS | 0.462 | 0.0035 | S |
| **Amodiaquine *vs* mefloquine** | 0.1529 | 0.3881 | NS | 0.3718 | 0.0234 | S | 0.0498 | 0.7794 | NS |
| **Amodiaquine *vs* quinine** | 0.3678 | 0.0251 | S | -0.0286 | 0.8661 | NS | -0.0597 | 0.7256 | NS |
| **Artesunate *vs* artemether** | 0.03048 | 0.8389 | NS | 0.1037 | 0.6143 | NS | 0.0757 | 0.6208 | NS |
| **Artesunate *vs* dihydroartemisinin** | -0.02102 | 0.8885 | NS | 0.0142 | 0.9427 | NS | 0.2573 | 0.109 | NS |
| **Artemether *vs* dihydroartemisinin** | 0.3442 | 0.0154 | S | 0.391 | 0.0245 | S | 0.146 | 0.3624 | NS |

S-statistically significant, NS- not statistically significant, ***vs*** - *versus*
